# Prognostic Significance of Immune Checkpoints HLA-G/ILT-2/4 and PD-L1 in Colorectal Cancer

**DOI:** 10.3389/fimmu.2021.679090

**Published:** 2021-05-13

**Authors:** Qiong-Yuan Chen, Yu-Xin Chen, Qiu-Yue Han, Jiang-Gang Zhang, Wen-Jun Zhou, Xia Zhang, Yao-Han Ye, Wei-Hua Yan, Aifen Lin

**Affiliations:** ^1^ Biological Resource Center, Taizhou Hospital of Zhejiang Province, Wenzhou Medical University, Linhai, China; ^2^ Alberta Institute, Wenzhou Medical University, Wenzhou, China; ^3^ Key Laboratory of Minimally Invasive Techniques & Rapid Rehabilitation of Digestive System Tumor of Zhejiang Province, Taizhou Hospital of Zhejiang Province, Wenzhou Medical University, Linhai, China; ^4^ Medical Research Center, Taizhou Hospital of Zhejiang Province, Wenzhou Medical University, Linhai, China

**Keywords:** HLA-G, ILT-2, ILT-4, PD-L1, colorectal cancer, prognosis

## Abstract

Immune checkpoint inhibitors (ICIs) have become a promising area of research for cancer treatment. In addition to the well-known ICIs targeting PD-1/PD-L1, HLA-G/ILT-2/-4 is relatively new immune checkpoint that has been evaluated in early clinical trials in patients with advanced solid tumors. In this study, the expression of HLA-G (n=157), ILT-2/4 (n=82), and PD-L1 (n=70) in epithelial cell adhesion molecule (EpCAM)-positive colorectal cancer (CRC) cells was analyzed by multicolor flow cytometry, and the prognostic significance of these molecules was evaluated. In EpCAM^+^ CRC cells, the median percentages of HLA-G, ILT-2, ILT-4, and PD-L1 were 14.90%, 67.70%, 8.55% and 80.30%, respectively. In addition, a positive correlation was observed between them (all *p*<0.001). Higher levels of these immune checkpoint proteins are associated with lymph node metastasis. In addition to the AJCC stage (*p*=0.001), Kaplan-Meier survival analysis showed that higher levels of HLA-G (*p*=0.041), ILT-2 (*p*=0.060), ILT-4 (*p*<0.001), PD-L1 (*p*=0.012), HLA-GILT4 (*p*<0.001) and ILT-2ILT-4 (*p*<0.001) were significantly associated with shorter survival of CRC patients. When CRC patients were stratified by early and advanced AJCC stages, HLA-G levels were only related to the survival among CRC patients with early disease stage (*p*=0.024), while ILT-4 levels were significant for both CRC patients with early (*p*=0.001) and advanced (*p*=0.020) disease stages. Multivariate cox regression analysis revealed that advanced AJCC stage (HR=2.435; *p*=0.005) and higher ILT-4 levels (HR=2.198; *p*=0.063) were independent risk factors for poor outcomes in patients with CRC. In summary, among the immune checkpoints, HLA-G/ILT-2/4 and PD-L1, ILT-4 is the most significant prognostic indicator of CRC. This finding indicated that a combination of immunotherapy strategies, such as ILT-4 blockade, could improve the clinical outcomes in patients with cancer. Moreover, multicolor flow cytometry can be employed as a reliable and efficient, alternative to immunohistochemistry, for evaluating the immune checkpoint proteins expressed in tumor lesions.

## Introduction

Immune checkpoint protein expression is one of the dominant mechanisms by which tumor cells evade the innate and adaptive anti-tumor immune responses and promote disease progression (1). Fortunately, blocking the immune checkpoint signaling pathway with immune checkpoint inhibitors (ICIs) has become a promising strategy in cancer treatment (2), which has shown remarkable outcomes in considerable proportion of patients with advanced solid tumors. The ICIs targeting the programmed death-1 (PD-1)/programmed death ligand-1 (PD-L1) axis have been developed and have marked a milestone in cancer immunotherapy (3). However, resistance to ICI treatment is common among many patients suffering from cancer. Either therapy with multiple combined ICIs or exploration of novel ICIs is necessary to overcome such resistance and ultimately improve the long-term clinical benefit (2).

HLA-G, a non-classical HLA class I antigen, is predominantly expressed in extravillous cytotrophoblasts during pregnancy, and under physiological conditions, its expression is restricted to a few immune-privileged tissues (4). However, an aberrant induction of HLA-G expression is observed in most cancer histological types, which is closely related to tumor metastasis and poor prognosis in clinical settings as well as in murine laboratory models (5). The expression of HLA-G, an immune tolerant and tumor promoting factor, has been extensively investigated, and its role as a novel immune checkpoint has been established (6, 7).

The immunosuppressive function of HLA-G is mediated by its interaction with immune inhibitory receptors, immunoglobulin-like transcript (ILT)-2 and/or ILT-4 (8). These receptors are expressed in tumor cells, as well as in a wide range of immune cells, such as B cells, T cells, natural killer cells (NK), dendritic cells (DCs), and myeloid-derived suppressor cells (MDSCs) (5). Interaction between tumor HLA-G and ILT-2/-4 expressed on the surface of immune cells can induce a broad spectrum of immune suppressive responses by (i) inhibiting T-cell proliferation and B-cell immunoglobulin production (9–11), (ii) impairing T-cell and NK-cell cytotoxicity, as well as maturation of DCs (12–14), (iii) promoting the proliferation of regulatory MDSCs and accumulation of M2-type macrophages, as well as inducing anergy by the generation of regulatory T cells (Treg), tolerant DC-10 cells, and invariant natural killer T cells (iNKTs) (15–19), and (iv) dampening the expression of chemokine receptors on B cells, T cells, and NK cells, thereby hampering their infiltration into the tumor microenvironment (20). In this context, the cytotoxic functions of tumor-infiltrating CD8^+^PD-L1-ILT-2^+^ cells could be selectively inhibited by tumor cell-expressing HLA-G (21). Moreover, ILT-2/-4 has been observed to be expressed in both solid tumors, such as stomach, lung, and liver cancers, as well as in hematological malignancies (22). Higher levels of ILT-2 expression have been found to be related to a shorter overall survival in patients with colon mucinous adenocarcinoma and gastric mixed adenocarcinoma. However, the level of ILT-2 expression was comparable among rectal mucinous adenocarcinomas and markedly lower in hepatocellular cancers than in that normal controls (23, 24). Additionally, previous studies have shown that the expression of ILT-4 is associated with tumor metastasis, advanced disease, and poor prognosis (25).

Currently, although immunohistochemistry is widely used in the evaluation of immune checkpoint protein expression in tumor lesions, many challenges, such as high inter-assay variability in routine performance remain to be addressed (26). In this study, a suspension of CRC cells was prepared from the frozen lesions and immune checkpoints, HLA-G, ILT-2, ILT-4, and PD-L1, were analyzed by multicolor flow cytometry gated using epithelial cell adhesion molecule (EpCAM); subsequently, the prognostic significance of the checkpoint proteins was evaluated.

## Materials and methods

### Patients With Colorectal Cancer

A total of 157 frozen primary CRC lesions from the Biological Resource Center, Taizhou Hospital of Zhejiang Province (National Human Genetic Resources Platform of China YCZYPT [2017]02) were included in the study. All patients with CRC were diagnosed and treated between June 2006 and May 2012 at Taizhou Hospital of Zhejiang Province, China. No immunotherapy was applied for these CRC patients. Written informed consent was obtained from each participant prior to surgery, and the study protocol was approved by the Ethics Review Board of Taizhou Hospital of Zhejiang Province (K20190766).

Detailed clinicopathological information was retrieved from the electronic medical records in the hospital. Among 157 patients with CRC (colon=90, rectal =67), 89 were male and 68 were female, with a median age of 69.5 years (range, 30 ~ 89 years). With respect to TNM staging, 10, 144, and 3 patients had pT_2_, T_3_ and T_4,_ respectively, and 75, 45, and 37 patients had pN_0_, N_1_, and N_2_, respectively. Similarly, 152 and 5 patients had pM_0_ and M_1_, respectively. AJCC disease stages I, II, III, and IV were found in 6, 68, 78, and 5 patients, respectively (AJCC 7th TNM staging system) (27).

Follow-up information was available for 82 of the 157 patients with CRC. The last follow-up was carried out in November 2016, with a median follow-up of 31 months, and 60 cancer-related events were recorded. Overall survival (OS) was determined from the date of surgery to the date of the event. The detailed clinicopathological characteristics of CRC patients are shown in **Table 1**.

**Table 1:** Association between high and low levels of immune checkpoint expression with clinical parameters in CRC patients.

Variables	No.	HLA-G (n=157; median=14.9%)			No.	ILT-2 (n=82; median=67.7%)			ILT-4 (n=82; median=8.55%)			No.	PD-L1 (n=70; median=80.30%)		
		Low	High	*p*		Low	High	*p*	Low	High	*p*		Low	High	*p*
Type															
colon	90	46 (51.1%)	44 (48.9%)	0.377	60	32 (53.3%)	28 (46.7%)	0.527	29 (48.3%)	31 (51.7%)	0.894	51	27 (52.9%)	24 (47.1%)	0.420
rectal	67	39 (58.2%)	28 (41.8%)		22	10 (45.5%)	12 (54.5%)		11 (50.0%)	11 (50.0%)		19	8 (42.1%)	11 (57.9%)	
Gender															
male	89	43 (48.3%)	46 (51.7%)	0.094	40	22 (55.0%)	18 (45.0%)	0.504	19 (47.5%)	21 (52.5%)	0.821	35	18 (51.4%)	17 (48.6%)	0.811
female	68	42 (61.8%)	26 (38.2%)		42	20 (47.6%)	22 (52.4%)		21 (50.0%)	21 (50.0%)		35	17 (48.6%)	18 (51.4%)	
Age															
<69.5yrs	82	43 (52.4%)	39 (47.6%)	0.655	41	17 (41.5%)	24 (58.5%)	0.077	19 (46.3%)	22 (53.7%)	0.579	35	15 (42.9%)	20 (57.1%)	0.232
>69.5 yrs	75	42 (56.0%)	33 (44.0%)		41	25 (61.0%)	16 (39.0%)		21 (51.2%)	19 (46.3%)		35	20 (57.1%)	15 (42.9%)	
pT stage															
T_2_	10	7 (70.0%)	3 (30.0%)	0.923	6	1 (16.7%)	5 (83.3%)	0.115	4 (66.7%)	2 (33.3%)	0.363	4	2 (50.0%)	2 (50.0%)	1.000
T_3+_T_4_	147	105 (71.4%)	42 (28.6%)		76	38 (50.0%)	38 (50.0%)		36 (47.4%)	40 (52.6%)		66	33 (50.0%)	33 (50.0%)	
pN stage															
N_0_	75	46 (61.3%)	29 (38.7%)	0.028	32	20 (62.5%)	12 (37.5%)	0.055	21 (65.4%)	11 (32.4%)	0.005	27	16 (59.3%)	11 (40.7%)	0.064
N_1_	45	26 (57.8%)	19 (42.2%)		27	15 (55.6%)	12 (44.4%)		13 (48.1%)	14 (51.9%)		22	13 (59.1%)	9 (40.9%)	
N_2_	37	13 (35.17%)	24 (64.9%)		23	7 (30.4%)	16 (69.4%)		6 (26.1%)	17 (73.9%)		21	6 (28.6%)	15 (71.4%)	
pM stage															
M_0_	152	39 (25.7%)	113 (74.3%)	0.088	78	40 (51.3%)	38 (48.7%)	0.960	39 (50.0%)	39 (50.0%)	0.329	66	33 (50.0%)	33 (50.0%)	1.000
M_1_	5	3 (60.0%)	2 (40.0%)		4	2 (50.0%)	2 (50.0%)		1 (25.0%)	3 (75.0%)		4	2 (50.0%)	2 (50.0%)	
AJCC stage															
I+II	74	45 (60.8%)	29 (39.2%)	0.113	32	20 (62.5%)	12 (37.5%)	0.102	21 (65.6%)	11 (34.4%)	0.015	27	16 (59.3%)	11 (40.7%)	0.220
III+IV	83	40 (48.2%)	43 (51.8%)		50	22 (44.0%)	28 (56.0%)		19 (38.0%)	31 (62.0%)		43	19 (44.2%)	24 (55.8%)	

### Sample Preparation

Single-cell suspensions from frozen CRC lesions were prepared by mechanical dissociation and enzymatic digestion. These lesions were kept at -80°C in an ultra-low temperature freezer at the Biological Resource Center, Taizhou Hospital of Zhejiang Province (National Human Genetic Resources Platform of China).

After taking them out from the freezer, the lesions were balanced at room temperature for 5 ~ 10 min, washed three times with HBSS buffer (Thermo Fisher Scientific, Grand Island, NY, USA), sectioned into small pieces (5 ~ 6 mm) and washed with HBSS buffer again. After dissociation, small lesion pieces were digested in HBSS buffer containing 2% fetal bovine serum (FBS) (Gibco, Grand Island, NY, USA), collagenase type IV (1 mg/ml, Thermo Fisher Scientific, Grand Island, NY, USA), and hyaluronidase (10 ng/mL, Merck, St. Louis, MO, USA) at 37°C for 4 h (28). After digestion, the cells were filtered with a 70 µm cell strainer (Corning, Durham, NC, USA) to obtain single cell suspension that was used for flow cytometry analysis.

### Flow Cytometry Analysis

To analyze HLA-G, ILT-2, ILT-4, and PD-L1 expression in CRC cells, monoclonal antibodies (mAbs), such as anti-EpCAM-FITC (CD326, Thermo Fisher Scientific, Carlsbad, CA, USA), anti-HLA-G-PE (MEM-G/09, Exbio, Vestec, Czech Republic), anti-ILT4-APC (CD85d, Thermo Fisher Scientific, Carlsbad, CA, USA), anti-ILT2-PE/Cy7 (CD85j, Biolegend, San Diego, CA, USA), and anti-PD-L1-PerCP/Cyanine 5.5 (Biolegend, San Diego, CA, USA), were used.

Flow cytometry analysis was performed using BD FACS Canto Plus (Becton Dickinson, San Jose, CA). Single cell suspensions (1×10^6^ ~ 5×10^6^) were resuspended in 100 µL PBS containing 2% FBS, and 5 µL of each of the aforementioned multicolor mAbs were added and incubated in the dark at 4°C for 30 min. For each mAb, a negative control, that is, an appropriate fluorescent-labeled isotype control, was used. Data were analyzed using FlowJo Jo™ software (Becton-Dickinson, San Jose, CA). CRC cells were identified as EpCAM (CD326)-positive cells.

### Statistical Analyses

Statistical analyses were performed using the statistical software package SPSS (version 13.0, SPSS, Inc. Chicago, IL, USA). Bivariate correlation analysis between HLA-G, ILT-2, ILT-4, and PD-L1 expression was performed using the Pearson test. The relation between the expression of HLA-G, ILT-2, ILT-4, and PD-L1 alone or combination in CRC cells and the clinicopathological status of patients with CRC was analyzed using the chi-square test. Survival analysis was performed using the Kaplan-Meier method (log-rank test). The significance of the appropriate variables for survival was evaluated using univariate and/or covariate Cox regression methods. Statistical significance was set at *p*<0.05 (Flowchart of the study protocol was shown in **Supplementary Figure 2** **)**.

## Results

### Expression of HLA-G, ILT-2, ILT-4 and PD-L1 in CRC Lesions

**Figure 1:**
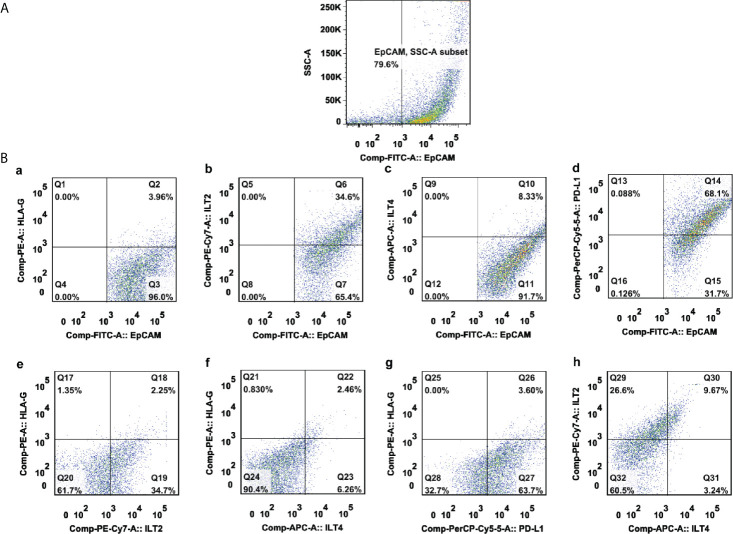
A representative multicolor flow cytometry analysis of either HLA-G, ILT-2, ILT-4, and PD-L1 alone or combination in a CRC lesion. **(A)** EpCAM**^+^**-gated CRC tumor cells; **(B)** expression percentage of **(a)** HLA-G, **(b)** ILT-2, **(c)** ILT-4, **(d)** PD-L1, **(e)** HLA-G:ILT-2, **(f)** HLA-G:ILT-4, **(g)** HLA-G:PD-L1, and **(h)** ILT-2:ILT-4 among EpCAM**^+^**-gated CRC tumor cells.

**Figure 2:**
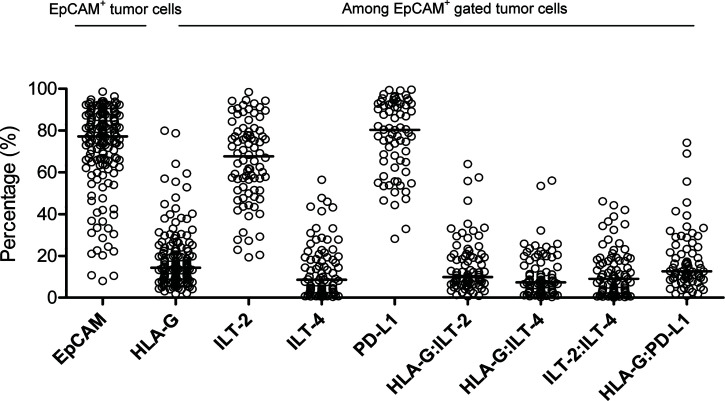
Percentage distribution of EpCAM+ CRC cells, as well as HLA-G, ILT-2, ILT-4, PD-L1, HLA-G:ILT-2, HLA-G:ILT-4, ILT2:ILT-4 and HLA-G:PD-L1 expression in EpCAM+ gated CRC cells.

Representative flow cytometry analysis is shown in **Figure 1**. Most samples (86.0%, 135/157) had more than 50% of EpCAM^+^ CRC cells. Among these samples, the median percentage of EpCAM (CD326)^+^ CRC cells was 77.2% (range: 7.6% ~ 98.60%), out of which the median percentage of the expression of HLA-G, ILT-2, ILT-4 and PD-L1 was 14.90% (n=157; range: 1.81% ~ 79.90%), 67.70% (n=82; range: 19.30% ~ 98.40%), 8.55% (n=82; range: 0.41% ~ 56.40%), and 80.30% (n=70; range: 28% ~ 99.60%), respectively. Moreover, the median percentage of the expression of HLA-G:ILT-2, HLA-G:ILT-4, ILT-2:ILT4 and HLA-G:PD-L1 was 9.90% (n=82; range: 0.61% ~ 64.0%), 7.35% (n=82; range: 0.26% ~ 56.0%), 8.99% (n=82; range: 0.01% ~ 46.10%), and 12.60% (n=70; range: 0.85% ~ 74.20%), respectively (**Figure 2** **)**.

Bivariate correlation analysis revealed a significant positive correlation between HLA-G, ILT-2, ILT-4, and PD-L1 expression in EpCAM**^+^** CRC cells. HLA-G and ILT-2 (r=0.463; *p*<0.001), HLA-G and ILT-4 (r=0.744; *p*<0.001), HLA-G and PD-L1 (r=0.573; *p*<0.001), ILT-2 and ILT-4 (r=0.408; *p*<0.001), ILT-2 and PD-L1 (r=0.603; *p*<0.001), and ILT-4 and PD-L1 (r=0.703; *p*<0.001) (**Supplementary Figure 1** **)**.

### Relationship Between HLA-G, ILT-2, ILT-4 and PD-L1 Expression and CRC Clinical Features

Based on the median levels of HLA-G, ILT-2, ILT-4, and PD-L1 expression, each of them was divided into high-and low-level expression groups. HLA-G^high^ and HLA-G^low^ groups had 72 and 85 cases, respectively, whereas ILT-2^high^ and ILT-2^low^ had 40 and 42 cases, respectively; ILT-4^high^ and ILT-4^low^ had 42 and 40 cases, respectively, and PD-L1^high^ and PD-L1^low^ had 35 and 35 cases, respectively (**Table 1** **)**.

Data showed that patients with CRC having distant lymph node metastasis (pT stage) were significantly associated with high levels of HLA-G (*p*=0.028), ILT-2 (*p*=0.055), ILT-4 (*p*=0.005), and PD-L1 (*p*=0.064). Moreover, high levels of HLA-G, ILT-2, ILT-4, and PD-L1 were more evident among patients in advanced disease stages. However, no marked difference was found between the HLA-G, ILT-2, ILT-4 and PD-L1 expression status and the clinicopathological parameters, such as patient age, sex, and colon or rectal cancer types (**Table 1** **)**.

### Levels of HLA-G, ILT-2, ILT-4 and PD-L1 Expression and CRC Survival

**Figure 3:**
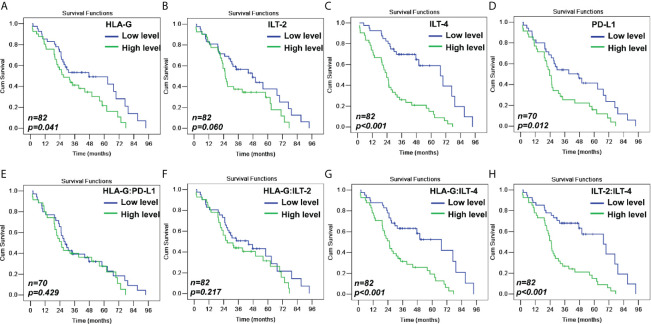
Kaplan-Meier survival analysis for patients with CRC between **(A)** HLA-G^high^ and HLA-G^low^ (*p*=0.041); **(B)** ILT-2^high^ and ILT-2^low^ (*p*=0.060); **(C)** ILT-4^high^ and ILT-4^low^ (*p*<0.001); **(D)** PD-L1^high^ and PD-L1^low^ (*p*=0.012); **(E)** HLA-G:PD-L1^high^ and HLA-G:PD-L1^low^ (*p*=0.429); **(F)** HLA-G:ILT-2^high^ and HLA-GILT-2^low^ (*p*=0.217); **(G)** HLA-GILT-4^high^ and HLA-G:ILT-4^low^ (*p*<0.001); and **(H)** ILT-2:ILT-4^high^ and ILT-2ILT-4^low^ (*p*<0.001).

In addition to the traditional pN (*p*<0.001), pM (*p*=0.004), and AJCC disease stage (*p*=0.001), Kaplan-Meier log-rank test results showed that higher expression of HLA-G, ILT-2, ILT-4, and PD-L1 was significantly associated with poor survival. A remarkably shorter overall survival (OS) for patients with CRC was observed between HLA-G^high^ and HLA-G^low^ (35.5 months *vs.* 48.7 months; *p*=0.041), ILT-2^high^ and ILT-2^low^ (36.1 months *vs.* 47.1 months; *p*=0.060), ILT-4^high^ and ILT-4^low^ (28.6 months *vs.* 58.1 months; *p*<0.001), and PD-L1^high^ and PD-L1^low^ (29.9 months *vs.* 45.5 months; *p*=0.012). Furthermore, patients with higher percentages of HLA-GILT-4 and ILT-2ILT-4 had significantly worse survival that those with lower HLA-GILT-4 and ILT-2ILT-4 expression (all *p*<0.001). No notable differences were found in CRC survival in terms of patient age, sex, pT stage, colon or rectal cancer types, and patients with low or high levels of HLA-G:ILT-2, and HLA-G:PD-L1 expression (**Table 2** and **Figure 3** **)**.

**Table 2:** Kaplan-Meier log-rank analysis of variables for CRC patient survival.

Variables		No. Total	No. Events	Survival (months) Mean (95% CI)	*p*
Subtypes	colon	60	42	42.4 (34.4 – 50.4)	0.528
	rectal	22	18	40.2 (29.0 – 51.4)	
Gender	male	40	32	43.0 (33.6-52.3)	0.444
	female	42	28	39.3 (37.5-47.1)	
Age	<69-year	41	30	43.3 (34.3-52.3)	0.563
	>69-year	41	30	39.6 (30.6-48.6)	
pT stage	T_2_	6	4	58.1 (37.1-79.2)	0.341
	T_3+4_	76	56	40.3 (33.7-46.9)	
pN stage	N_0_	32	18	56.6 (45.5-67.7)	<0.001
	N_1_	27	22	40.4 (30.8-49.9)	
	N_2_	23	20	23.7 (15.5-31.8)	
pM stage	M_0_	78	56	42.9 (36.4-49.4)	0.004
	M_1_	4	4	16.5 (6.30-26.7)	
AJCC stage	I+II	32	18	56.7 (45.5-67.7)	0.001
	III+IV	50	42	32.7 (25.9-35.6)	
Percentage (%)*					
HLA-G	Low *vs.* high	41	27	48.7 (38.7-58.6)	0.041
		41	33	35.5 (27.8-43.2)	
HLA-G:PD-L1		35	30	39.0 (29.4- 48.6)	0.429
		35	29	35.5 (26.9- 44.2)	
ILT-2		42	28	47.1 (37.3-56.9)	0.060
		40	32	36.1 (28.1-44.0)	
ILT-4		40	20	58.1 (48.0-68.2)	<0.001
		42	40	28.6 (21.9-35.4)	
PD-L1		35	26	45.5 (35.1-55.9)	0.012
		35	33	29.9 (22.3-37.4)	
HLA-G:ILT-2		41	28	45.1 (35.1-55.0)	0.217
		41	32	38.5 (30.2-46.8)	
HLA-G:ILT-4		41	22	54.7 (44.0-65.4)	<0.001
		41	38	31.6 (24.4-38.8)	
ILT-2:ILT-4		41	21	56.0 (45.5-66.5)	<0.001
		41	39	30.0 (23.4-36.6)	

*Percentage among EpCAM+ gated CRC tumor cells.

**Figure 4:**
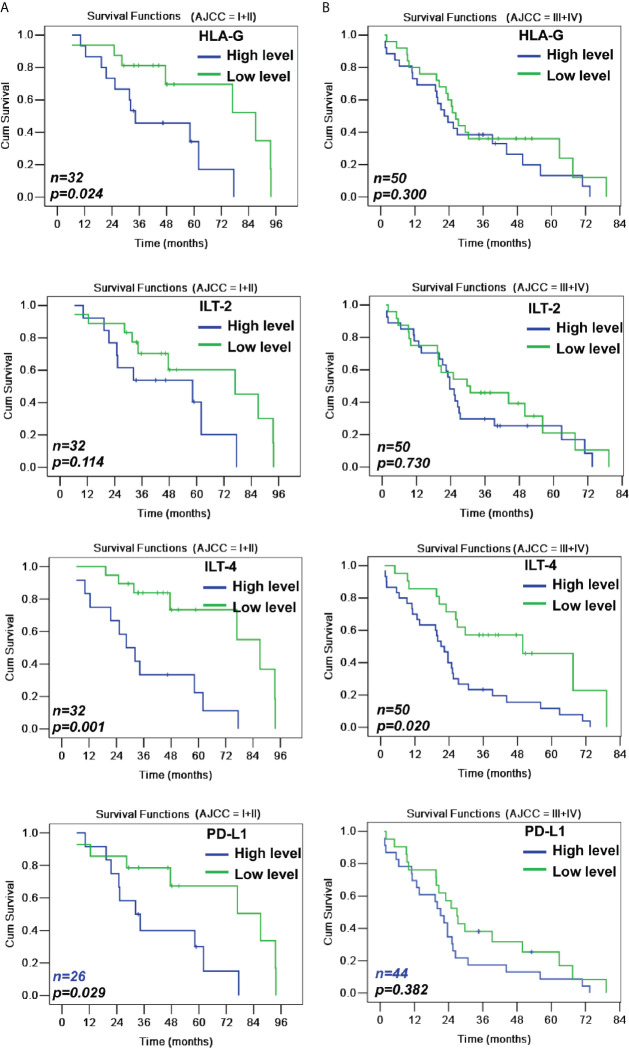
Kaplan-Meier survival analysis of low and high levels of HLA-G, ILT-2, ILT-4 and PD-L1 for survival either in AJCC stage I+II **(A)** or in AJCC stage III+IV **(B)** CRC patients.

When CRC patients were stratified by early and advanced AJCC stages, HLA-G levels were only related to the survival among CRC patients with early disease stage (43.4 months *vs.* 70.4 months; *p*=0.024), while ILT-4 levels were significant for both CRC patients with early (36.2 months *vs.* 73.6 months; *p*=0.001) and advanced (25.5 months *vs.* 47.4 months; *p*=0.020) disease stages (**Table 3** and **Figure 4**).

**Table 3:** Log-rank Mantel-Cox analysis of stratified variables in survival by tumor AJCC status in CRC patients.

Variables	Stratified variables	AJCC I+II			*p*	AJCC III+IV			*p*
		No. Total	No. Events	Survival Mean (95% CI)		No. Total	No. Events	Survival Mean (95% CI)	
HLA-G	low	16	8	70.4 (53.9 – 87.0)	0.024	25	19	37.2 (26.5 – 47.9)	0.300
	high	15	11	43.4 (30.5 – 56.2)		26	22	30.4 (21.1 – 39.7)	
ILT-2	low	18	10	64.7 (48.3 – 81.1)	0.114	24	18	36.7 (25.8 – 47.5)	0.730
	high	13	9	46.3 (32.1 – 60.4)		27	23	31.3 (22.0 – 40.5)	
ILT-4	low	19	8	73.6 (59.3 – 87.8)	0.001	21	12	47.4 (34.5 – 60.3)	0.020
	high	12	11	36.2 (22.4 – 50.0)		30	29	25.5 (18.0 – 33.0)	
PD-L1	low	14	8	67.9 (49.4 – 86.3)	0.029	21	18	34.2 (23.4 – 45.0)	0.382
	high	12	10	41.3 (27.5 – 55.0)		23	23	23.8 (15.6 – 32.1)	

### Prognostic Value of HLA-G, ILT-2, ILT-4 and PD-L1 Expression in Patients With CRC

Finally, using univariate and covariate Cox regression analyses, we analyzed the prognostic value of AJCC disease stage and HLA-G, ILT-2, ILT-4 and PD-L1 expression, respectively and evaluated how these variables were significantly related to the survival of patients with CRC. Although AJCC disease stage, HLA-G, ILT-2, ILT-4, and PD-L1 expression were closely related to the survival of patients with CRC, covariate Cox regression data revealed that only AJCC disease stage (HR=2.435, 95% CI: 1.300 ~ 4.562; *p*=0.005) and ILT-4 (HR=2.198, 0.985 ~ 5.042; *p*=0.063) were independent prognostic factors for those patients (**Table 4**).

**Table 4:** Cox regression analysis for prognostic value of variables for CRC patients.

Variables	Categories	Univariate Analysis		Multivariate Analysis	
		HR (95% CI)	*P*	HR (95% CI)	*P*
AJCC stage	III/IV *vs.* I/II	2.694 (1.502-4.831)	0.001	2.435 (1.300-4.562)	0.005
HLA-G	high *vs.* low	1.727 (1.016-2.936)	0.043	0.859 (0.428-1.723)	0.668
ILT-2		1.652 (0.974-2.801)	0.062	0.933 (0.488-1.783)	0.834
ILT-4		3.537 (1.955-6.272)	<0.001	2.198 (0.985-5.042)	0.063
PD-L1		1.962 (1.146-3.360)	0.014	1.224 (0.569-2.631)	0.605

HR, hazard ratio; 95% CI, 95% confidence interval.

## Discussion

Considerable therapeutic success has been achieved by using ICIs, such as clinically validated antibodies targeting the PD-1/PD-L1 and cytotoxic T-lymphocyte-associated protein 4 (CTLA-4)/B7 signaling pathways. Exploration of novel immune checkpoints and development of new ICIs has gained momentum in the field of cancer immunotherapy (29). Among them, the HLA-G/ILTs signaling pathway has been recently recognized as a new immune checkpoint, and ICIs targeting this interaction are in early clinical trials to treat different types of advanced solid tumors (30).

EpCAM-based cell surface markers for identification of epithelial cancers and as a target for circulating tumor cell (CTC) isolation and enrichment have long been established (31). In this study, the percentage of EpCAM^+^ CRC cells varied, but most samples (86.0%, 135/137) had more than 50% of these cells. In a study with 21 fresh CRC lesions that were analyzed using a protocol similar to ours, Satio et al. (32) reported that both low and high counts of EpCAM^+^ cells detected by flow cytometry in cell suspensions isolated from the lesions were tumor cells of a proportion of 64.0%±15.8% as compared to 72.1±20.0% observed in our study. This marginal difference might be attributed to the different compositions of the CRC cohorts analyzed.

We then analyzed the expression of immune checkpoint proteins, HLA-G, ILT-2, ILT-4, and PD-L1, using multicolor flow cytometry in EpCAM^+^-gated tumor cells from frozen CRC samples. Our results revealed that in samples with higher counts of EpCAM^+^ tumor cells, the expression of HLA-G, ILT-2, ILT-4, and PD-L1 was significantly related to distant lymph node metastasis, advanced disease stage, and shorter patient survival. To be noted, when CRC patients were stratified by early and advanced AJCC stages, our data showed that HLA-G levels were only related to the survival in CRC patients with early disease stage, and ILT-4 levels were significant for both CRC patients with early and advanced disease stages. In addition to well-known and clinically proven ICIs targeting the PD-1/PD-L1 signaling pathway, it has been demonstrated using the Cox regression data that a higher ILT-4 expression could be an independent prognostic risk factor for patients with CRC. Phase I clinical trials based on the HLA-G/ILTs interaction blockade for the treatment of different types of advanced solid tumors have been performed, and the clinical benefits are yet to be evaluated (30).

The expression of ILT-2 and ILT-4 has been observed in various immune and tumor cells (22). Several studies have demonstrated that the binding of HLA-G expressed on tumor cells to immune cells bearing ILT-2 and ILT-4 aids the evasion of the anti-tumor immune responses of the host. This interaction can suppress both the peripheral and intra-tumor functions of immune-competent cells by (i) inhibiting the proliferation and cytolytic functions of T cells and NK cells, thereby hampering the maturation and antigen-presentation ability of DCs, and (ii) inducing the generation of immune regulatory cells, such as Tregs, MDSCs, DC-10 and M2 type macrophages (5). In this context, our findings in this study support the conclusion drawn from previous studies that tumor HLA-G expression is closely related to tumor progression and poor clinical outcomes in patients with cancer. A large-scale genome-wide association (GWAS) study of East Asians (22,775 CRC patients and 47,731 controls) revealed that *HLA-G* is one of the leading loci associated with the risk of CRC (33). At the protein level, enhanced HLA-G expression has been found to be greatly correlated with weak anti-tumor immune response, disease progression, and poor survival, and HLA-G is an independent prognostic predictor of CRC (34, 35).

The association of ILT-2 and ILT-4 expression with tumor malignancy and poor prognosis has been addressed in many previous reports (25). Pre-clinical data have shown that ILT-2/4 blockade can remarkably restore the cytotoxic capacity of CD8+ T cells or NK cells against tumor cells, thereby promoting antitumor immune responses (36–38). We found that the expression of ILT-2 and ILT-4 in CRC lesions was significantly correlated with HLA-G and PD-L1 expression. It has been previously reported that the counts of antigen-presenting cells and NK cells, and the expression of ILT-2/4 on T cell surface were increased by HLA-G (39). Moreover, there has been evidence that HLA-G upregulates ILT-4 expression in CRC cell lines in an autocrine manner, and HLA-G/ILT-4 engagement enhances CRC cell proliferation, migration, and invasion by the activation of protein kinase B (AKT)/extracellular signal regulated kinase (ERK) signaling (40). HLA-G/ILT-4 engagement *via* ERK signaling may be involved in the upregulation of vascular endothelial growth factor C (VEGF-C) expression in non-small cell lung cancer (NSCLC) cells (41). García et al. (42) reported more evidence regarding the mechanism by which HLA-G/ILT-4 interaction increased VEGF-C expression in clear cell renal cell carcinoma. It indicated the significance of HLA-G/ILT-4 signaling in the promotion of angiogenesis during tumor development, which is the rationale behind the findings of Carosella et al. (43) who proposed that a combination of anti-angiogenic therapies can enhance the synergistic antitumor activity of ICI monotherapy.

In summary, our findings revealed that ILT-4 is the most significant prognostic indicator among HLA-G/ILT-2/4 and PD-L1 immune checkpoint proteins in patients with CRC. Combinatorial immunotherapy with different ICIs could improve the clinical outcomes in patients with cancer. To be noted, ICIs such as PD1 antibody are only approved for solid cancer patients with microsatellite instability-high (MSI-H), mismatch-repair deficiency (dMMR) or high tumor mutational burden (TMB-high). Though MSI-H, dMMR or TMB-high molecular phenotype varies among cancer patients, and not all patients with the MSI-H, dMMR or TMB-high phenotype respond to the ICIs therapy. In this context, exploration of mechanisms underlying resistance to ICIs is necessary (44, 45). Moreover, due to many challenges, such as high variation in inter-assay precision and cut-off values associated with routine immunohistochemistry, multicolor flow cytometry can serve as a reliable and efficient alternative method. However, due to these CRC samples were collected and frozen between 2006 and 2012, no case-matched fresh samples were analyzed for the expression of HLA-G, ILT-2, ILT-4 and PD-L1 with flow cytometry method. Whether cryopreservation with different time could affect the expression of these molecules remains to be evaluated in our future prospective studies.

## Data Availability Statement 

The original contributions presented in the study are included in the article/**Supplementary Material**. Further inquiries can be directed to the corresponding authors.

## Ethics Statement

The studies involving human participants were reviewed and approved by Ethics Review Board of Taizhou Hospital of Zhejiang Province (K20190766). The patients/participants provided their written informed consent to participate in this study.

## Author Contributions 

Concept and design, manuscript drafting and statistical analysis: W-HY and AL. Experiments and methods: Q-YC and Y-XC. Data collection and interpretation of data: Q-YH, J-GZ, W-JZ, XZ, and Y-HY. All authors contributed to the article and approved the submitted version.

## Funding

This work was supported by grants from Science and Technology Bureau of Taizhou (1901ky01; 1901ky04; 20ywa01; 20ywa03).

## Conflict of Interest

The authors declare that the research was conducted in the absence of any commercial or financial relationships that could be construed as a potential conflict of interest.

## Supplementary Material

Supplementary Figure 1
**|** Bivariate correlation analysis between the expression of HLA-G, ILT-2, ILT-4, and PD-L1 performed using Pearson test.

Supplementary Figure 2 Flowchart of the study protocol.
