# Supplementary figures and images for: Prognostic Significance of Immune Checkpoints HLA-G/ILT-2/4 and PD-L1 in Colorectal Cancer
Source: Front Immunol. 2021 May 13;12:679090. doi: 10.3389/fimmu.2021.679090 (PMC8155601; doi:10.3389/fimmu.2021.679090)

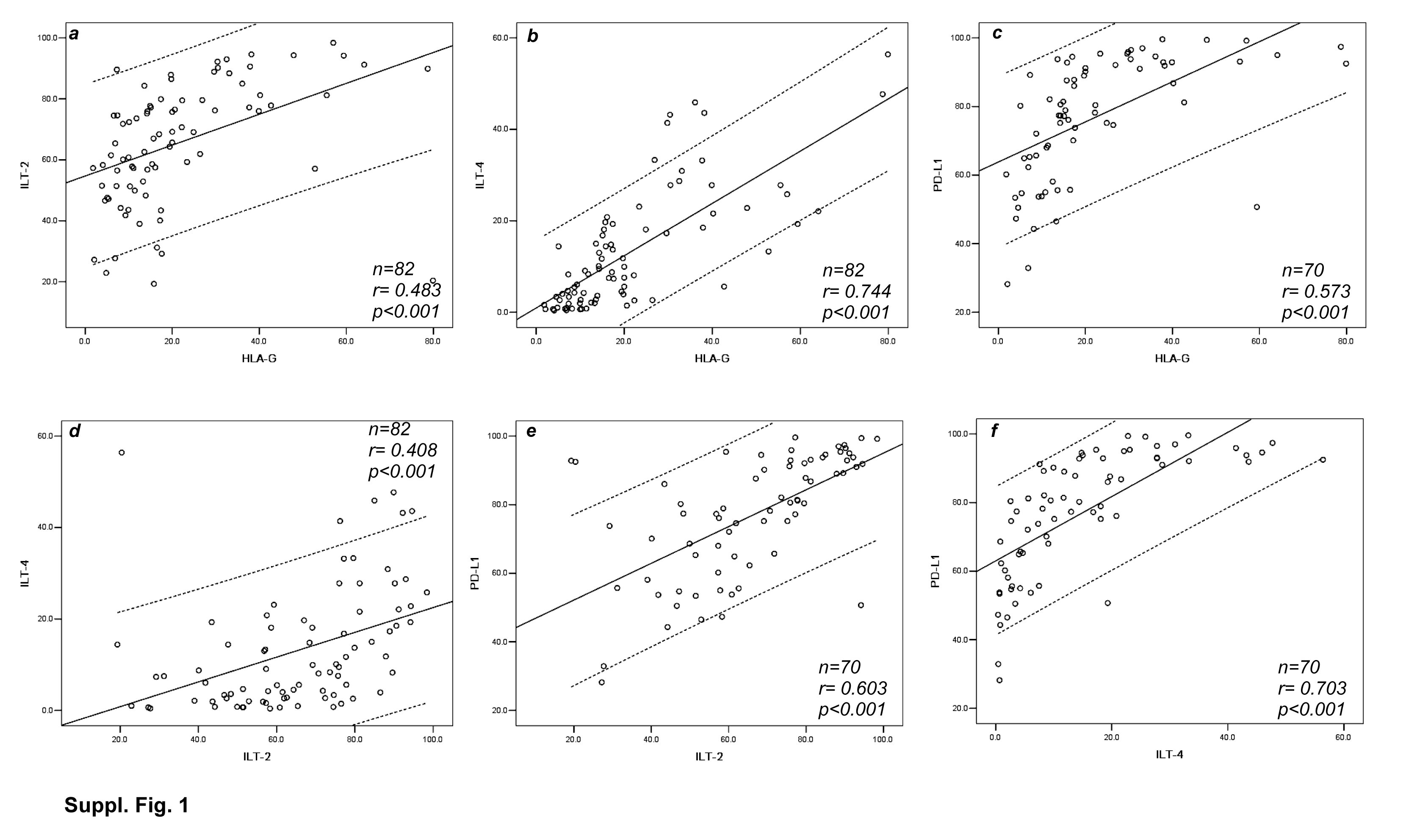

Supplement: Supplementary Figure 1 — | Bivariate correlation analysis between the expression of HLA-G, ILT-2, ILT-4, and PD-L1 performed using Pearson test. [file Image_1.tif]

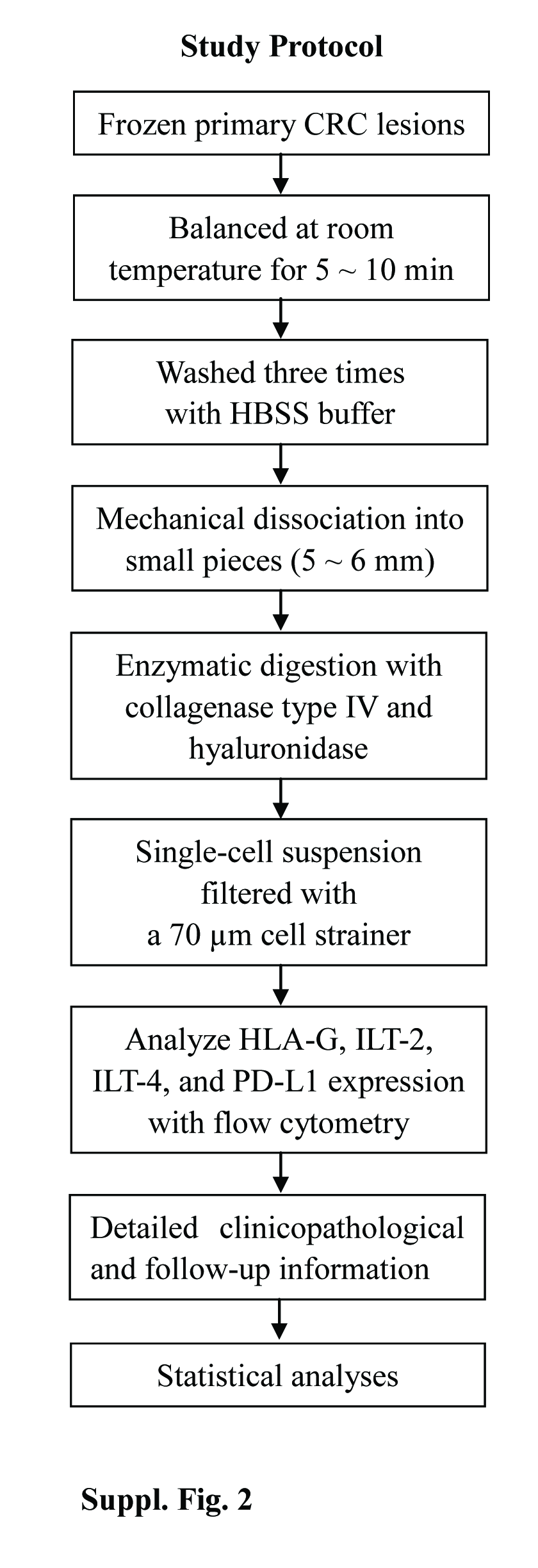

Supplement: Supplementary Figure 2 — Flowchart of the study protocol. [file Image_2.tif]
